# Analysis of Carbon Emission Projections and Reduction Potential of Resource-Dependent Urban Agglomerations from the Perspective of Multiple Scenarios—A Case Study of Hu-Bao-O-Yu Urban Agglomeration

**DOI:** 10.3390/ijerph20054250

**Published:** 2023-02-27

**Authors:** Xuanwei Ning, Yushuang He, Jiayi Zhang, Chengliang Wu, Yang Zhang

**Affiliations:** School of Economics and Management, Beijing Forestry University, Beijing 100083, China

**Keywords:** peak carbon dioxide emission, carbon emission reduction, scenario prediction, system dynamics, urban agglomeration

## Abstract

The Hu-Bao-O-Yu urban agglomeration is an important energy exporting and high-end chemical base in China, and is an important source of carbon emissions in China. The early achievement of peak carbon emissions in this region is particularly crucial to achieving the national carbon emission reduction targets. However, there is a lack of multi-factor system dynamics analysis of resource-dependent urban agglomerations in Northwest China, as most studies have focused on single or static aspects of developed urban agglomerations. This paper analyses the relationship between carbon emissions and their influencing factors, constructs a carbon emission system dynamics model for the Hu-Bao-O-Yu urban agglomeration, and sets up different single regulation and comprehensive regulation scenarios to simulate and predict the carbon peak time, peak value, and emission reduction potential of each city and urban agglomeration under different scenarios. The results show that: (1) Hohhot and Baotou are expected to reach peak carbon by 2033 and 2031 respectively, under the baseline scenario, while other regions and the urban agglomeration will not be able to reach peak carbon by 2035. (2) Under single regulation scenarios, the effect of factors other than the energy consumption varies across cities, but the energy consumption and environmental protection input are the main factors affecting carbon emissions in the urban agglomeration. (3) A combination of the economic growth, industrial structure, energy policy, environmental protection, and technology investment is the best measure to achieve carbon peaking and enhance the carbon emission reduction in each region as soon as possible. In the future, we need to coordinate the economic development, energy structure optimisation and transformation, low-carbon transformation of industry, strengthen research on carbon sequestration technology, and further increase the investment in environmental protection to make the Hu-Bao-O-Yu urban agglomeration a resource-saving urban agglomeration with an optimal emission reduction.

## 1. Introduction

Global climate change has become a growing concern for the international community, and the IPCC has indicated that more than 90% of climate change is anthropogenic in origin [1,2]. The current exponential growth in greenhouse gas emissions from human activities, especially the increase in CO_2_ concentration, has already outweighed the negative environmental impacts of natural variability [3,4]. Although the novel coronavirus pneumonia outbreak in late 2019 cut global carbon emission levels to some extent [5], carbon emissions began to rebound after the outbreak along with economic recovery. Therefore, the contribution of carbon emissions to the greenhouse effect and how to reduce them have become a hot topic of research, and carbon peaking and carbon neutrality have become the key to combating climate change.

As the world’s top carbon emitter, the peaking time, level of carbon attainment, and the time of neutralisation in China have become the focus of global attention [6,7]. In order to achieve the “double carbon” target, governments at all levels have gradually focused on the relationship between carbon peaking and carbon neutrality, concluding that it is necessary to peak as early as possible to reduce the pressure of carbon neutrality and to achieve carbon peaking at a lower cost on the other hand [8]. Therefore, predicting the time and peak of carbon emission attainment and modelling the carbon reduction pathway become the basis for formulating carbon reduction policies. Due to the large differences in economic development and environmental protection efforts among cities and urban agglomerations, a laddering approach is necessary to achieve carbon peaking in China as a whole [9]. The resource-dependent urban agglomerations in the west are characterised by a changing cycle of resource exploitation and processing, with non-renewable energy resources accounting for more than 90% of the total [10], while low-level low-carbon industries in urban agglomerations lack corresponding low-carbon technology research and application services [11]. How to reduce carbon emissions in such urban agglomerations is an important challenge for China to achieve the target of “double carbon”. Therefore, it is an important issue for further research to predict the peak time and peak value of carbon emissions in typical western resource-dependent urban agglomerations, and to identify the key factors influencing carbon emissions in such urban agglomerations, so as to formulate a more efficient carbon emission reduction plan.

The Hu-Bao-O-Yu urban agglomeration is located at the northern end of the Bao-Kun Passage in China’s “two horizontal and three vertical” urbanisation development pattern, and is an important hub connecting Northwest China, North China, and Northeast China [12]. The urban agglomeration is rich in energy and mineral resources and has many high-energy-consuming industries. It is an important energy exporter and high-level chemical base in China, and is an important area as a source of carbon emissions in China. The simulation of carbon peaking in this city cluster, the identification of key factors influencing its carbon emissions, and the exploration of how to reduce carbon peaking with high quality and implement carbon emission reduction efficiently are of reference value for other resource-dependent urban agglomerations in western China and even for the whole country. Therefore, by constructing a carbon emission system dynamics model for urban agglomerations, this paper predicts the peaking times and peaks of energy carbon emissions in Hohhot, Baotou, Ordos, Yulin, and the urban agglomeration under different scenarios, provides insight into the dynamics and feedback structures within and between subsystems, predicts the future development trend of carbon emissions, and sets up different scenarios to simulate the impact of different scenarios on the carbon peaks of urban agglomerations, so as to provide a theoretical reference for achieving the carbon peaks in Hohhot, Baotou, Ordos, and Yulin as scheduled.

This paper is broken down into the following sequence: part II introduces the literature review of previous studies on the carbon peak time prediction and carbon reduction pathways; part III introduces the research area and research methodology of this paper; part IV establishes the carbon emission system model of urban agglomeration using the basic data of each city; part V regulates different variables of the model, analyses the carbon peak time and carbon peak volume of urban agglomerations under different scenarios, and compares the advantages and disadvantages of various peak strategies; part VI presents the conclusions drawn and the policy recommendations formulated in this paper; and finally, there is the future outlook of this paper.

## 2. Literature Review

### 2.1. Carbon Peak Forecast

Previous studies on carbon peaking in China have mostly been oriented towards future peak targets, using methods such as the STIRPAT model [13,14], the IPAT model [15], the China Integrated Energy and Environmental Policy Evaluation Model [16], the CGE model [17], the BP neural network combination model [18], the Environmental Kuznets curve projections [19,20], and Monte Carlo dynamic simulations [21], to predict national and provincial peak carbon trends by modeling future changes in different variables. At the national level, Sun et al. [22] estimated China’s peak GHG emissions based on scenario analysis and the STIRPAT model; Hu and Dong [17] studied the problems in achieving China’s peak carbon target based on an extended CGE model. At the city level, Cai and Wu [23] used the Grey Gompertz model to predict the peak carbon emissions of 16 provinces in China and found that three provinces, Inner Mongolia, Xinjiang, and Ningxia, would not reach their peak carbon emissions by 2032. Zhang et al. [24] projected that the pilot low-carbon city of Baoding would reach peak carbon emissions in 2024 based on a BP model by optimising the population size, reducing the share of coal consumption, and increasing the growth rate of tertiary industry GDP. The combination of previous studies shows that 2030 is the most appropriate time for China’s overall carbon peak, given the dual requirements of safeguarding economic development and weakening carbon emissions. However, there are significant differences in the time to reach the peak between regions. Economically developed and technologically advanced regions will be the first to reach the peak. For example, the peaking time for the southeast coast and the three major urban agglomerations will be advanced to around 2025 [25,26], while the peaking time for the central and western regions will be delayed to 2025–2030, or even after 2030 [26].

### 2.2. Carbon Reduction Path

Carbon emission peak predictions can be used to determine the future trend of carbon emissions. The further analysis of carbon emission scenarios can help to explore the potential impact of different policies and scenarios on the future trend of carbon emissions, and thus quantify the path to achieve future carbon reduction targets. Research on the carbon pathways of representative cities can be valuable for other cities and even for the whole country. Tian et al. [27] used the generalised Dee-style exponential decomposition method to find that economic scale is the primary factor leading to the increase in carbon emissions in the Yangtze River Economic Zone region, that the energy consumption intensity and output carbon intensity are important factors inhibiting the increase in carbon emissions, and that technological progress is a key factor promoting carbon emission reduction. Jan et al. [28] used the extended STIRPAT model and the carbon emission potential index model to find that the main factors affecting industrial carbon emissions in northern Jiangsu are the energy consumption intensity, industrial economic scale, and industry structure. The path to peak carbon research at the national level is similar to that of cities. It is mainly based on the analysis of the energy mix, technological progress, and economic growth. Qi et al. [29] suggest that although controlling the carbon intensity of the energy system and the share of non-fossil energy sources is crucial to reach the carbon peak, energy efficiency and technological progress are more effective in achieving the peak. Wang et al. [30] argue that high-quality economic development has a greater impact on the reduction in CO_2_ emissions, with rapid GDP growth promoting the investment in technology and technological progress achieving carbon reductions. Shan et al. [31] used the LEAP model to simulate the initial and final energy demands in 2020 and 2030 under different scenarios, and found that the initial energy demand was 4840–5070 GT in 2020 and 5580–5870 GT in 2030, and that a 40–45% reduction in the carbon emission intensity could be achieved under all development scenarios.

### 2.3. System Dynamics

The system dynamics approach was first proposed by the American scientist Forrester. It is a quantitative approach based on the feedback control theory and uses numerical simulation to study complex socio-economic systems, allowing for system analysis and forecasting in the medium and long term [32,33]. This method can explain the behaviour patterns of the system determined by the feedback mechanism among the elements within the system, simulate the dynamic development of complex systems such as the urbanisation development system [34] and air pollution system [35], and further improve the structure and function of the system based on this [36,37]. System dynamics is an integrated simulation and prediction model, the greatest advantage of which is that it can avoid the one-sidedness of low-dimensional and single predictions and is suitable for dealing with non-linear and long-term problems of more complex systems. It has been widely used in the study and management of energy consumption, greenhouse gas emissions, and environmental management at different scales, including the national, regional, and industrial scales [38,39,40].

### 2.4. Research Gaps

Current research has provided a wide range of ideas and directions for the modeling of peak carbon scenarios and predictions of emission reduction potential.

However, the prediction of carbon peaking is a dynamic and complex system problem involving economic, demographic, energy, and technology factors. Existing studies on carbon emission pathways are mostly focused on local qualitative research, mostly at a static or single level, and lack a systematic dynamic analysis of multiple factors. Few scholars have considered every aspect of carbon emissions comprehensively from a systems science perspective, thus making it difficult to grasp the constraints and objective laws in the process of carbon neutrality as a whole. 

At the same time, due to the uneven development of resource endowment as well as socio-economic, industrial, and agricultural development in different regions of China, ladder peaking is more suitable for the actual needs of carbon peaking in China. While the existing research scales are mostly national or provincial, systematic studies on urban agglomerations focus on economically developed regions such as Beijing-Tianjin-Hebei, Yangtze River Delta, and Pearl River Delta, and few scholars have focused on western urban agglomerations that rely on energy development pathways. However, the uneven and non-synchronous nature of carbon peaks determines that the energy carbon peaks of resource-dependent urban agglomerations in the west will be an important part of China’s carbon peaks. Therefore, the use of relevant research methods to analyse the energy carbon peak reduction pathways of such urban agglomerations is of great significance to the achievement of China’s carbon reduction targets.

In the Yellow River basin, the Hu-Bao-O-Yu urban agglomeration accounts for 16% of the carbon emissions and 24.8 t of carbon emissions per capita, which is a large share of carbon emissions. The development of a carbon peaking action plan for the Hu-Bao-O-Yu urban agglomeration under the ladder peaking route must take into account a variety of factors including the historical regional carbon emissions and economic and social development. Based on this, this paper uses a system dynamics approach to construct a carbon emission system for the Hu-Bao-O-Yu urban agglomeration, which includes six subsystems: economy, transportation, energy, population, and carbon sink, with the aim of comprehensively exploring the key factors affecting carbon emission changes in this urban agglomeration and predicting the peak time and peak value of carbon in the urban agglomeration under different scenarios.

The first innovation of this paper is to simulate and optimise peak carbon pathways for a typical western resource-dependent urban agglomeration, which fills the gaps of previous studies. This study will provide recommendations on the energy policy, economic development, and environmental protection input for the Hu-Bao-O-Yu urban agglomeration to achieve the peak carbon target, and also provide references for the peak carbon planning paths of other western resource-dependent urban agglomerations.

The second innovation of this paper is the systematic and theoretical overall analysis of carbon emission in the Hu-Bao-O-Yu urban agglomeration by applying the system theory thinking method. By constructing a system dynamics simulation model covering the organic whole consisting of an economic subsystem, transportation subsystem, energy subsystem, population subsystem and carbon sink subsystem, it overcomes the problem of simple reductionist thinking in studying this kind of problem by simply fragmenting the simulation of carbon emissions of urban agglomeration, which is conducive to exploring the best path to improving the quality of the carbon peak in the Hu-Bao-O-Yu urban agglomeration.

## 3. Materials and Methods

### 3.1. Study Area

**Figure 1:**
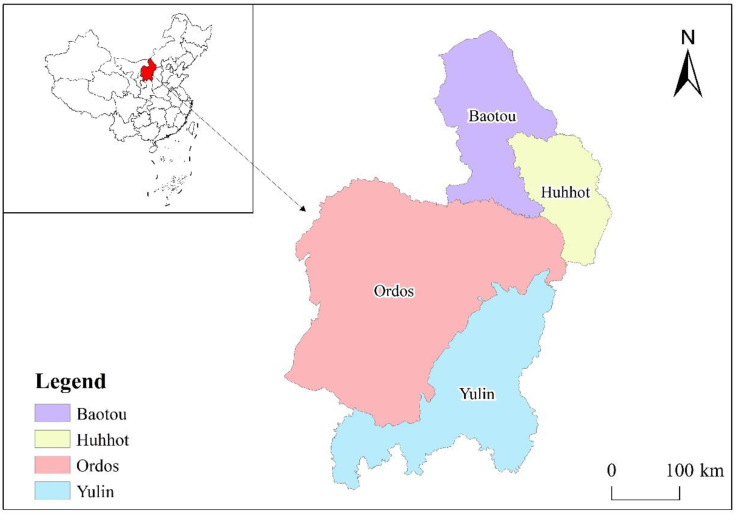
Location of the Hu-Bao-O-Yu urban agglomeration.

The Hu-Bao-O-Yu urban agglomeration is located in the central part of the northern dry zone of China (36°48′50″~42°44′5″ N, 106°28′16″~112°18′7″ E), in the interlocking zone of agriculture and livestock [12], and is a city cluster with Hohhot, Baotou, Ordos, and Yulin. Hohhot is the core city, while Baotou, Ordos, and Yulin are important node cities in the region (Figure 1). With a land area of about 175,000 km^2^ and a total population of about 10,404,000, the Hu-Bao-O-Yu urban agglomeration is an important energy and coal chemical base, an agricultural and pastoral products processing industry base, and a rare earth new materials industry base in China, as well as an important ecological barrier in the western region of China and a pioneer area for urban–rural integration and development in ethnic areas, with a huge potential for complementary resources and cooperative utilisation between the cities [41,42]. Since 2000, the intensive promotion of the western development and the remarkable achievements of the “Belt and Road” construction have provided strong support for the urban agglomeration to enhance its development and expand its opening up, making the Hu-Bao-O-Yu urban agglomeration experience a rapid urban expansion. However, the rapid urban expansion has led to increasingly prominent problems such as an imbalance in the economic structure and damage to resources and the environment, coupled with the low threshold of ecological risk resistance and relatively low level of socio-economic development in this urban agglomeration, which has had a serious impact on CO_2_ emission reduction projects in the region [43].

### 3.2. Research Methods

#### 3.2.1. Carbon Emission Factor Method

The carbon emission factor method is currently the most widely used method for measuring carbon emissions [44]. This method is simple and accurate to calculate. According to the IPCC, the carbon dioxide emissions for each component are equal to the activity data multiplied by the emission factor [45]. We calculated the carbon emissions in the Hu-Bao-O-Yu region based on the primary energy consumption in each region of urban agglomeration and the carbon emission factors in the 2006 IPCC Guidelines for National Greenhouse Gas Inventories. The standard coal conversion factors and carbon emission factors are shown in Table 1.

The formula for calculating carbon emissions in the urban agglomeration is shown below:

$$C=\sum C_{i}=\overset{8}{\underset{i=1}{\sum }}E_{i}\times \delta_{i}\times \gamma_{i}\times 44/12$$

In the formula, C represents carbon emissions (million t) in that year; i is the type of primary energy consumption; $E_{i}$ represents the energy consumption of the ith energy source; $\delta_{i}$ represents the converted standard coal factor of the ith energy source; and $\gamma_{i}$ represents the carbon emission factor of the ith energy source.

#### 3.2.2. System Dynamics Simulation Method

**Figure 2:**
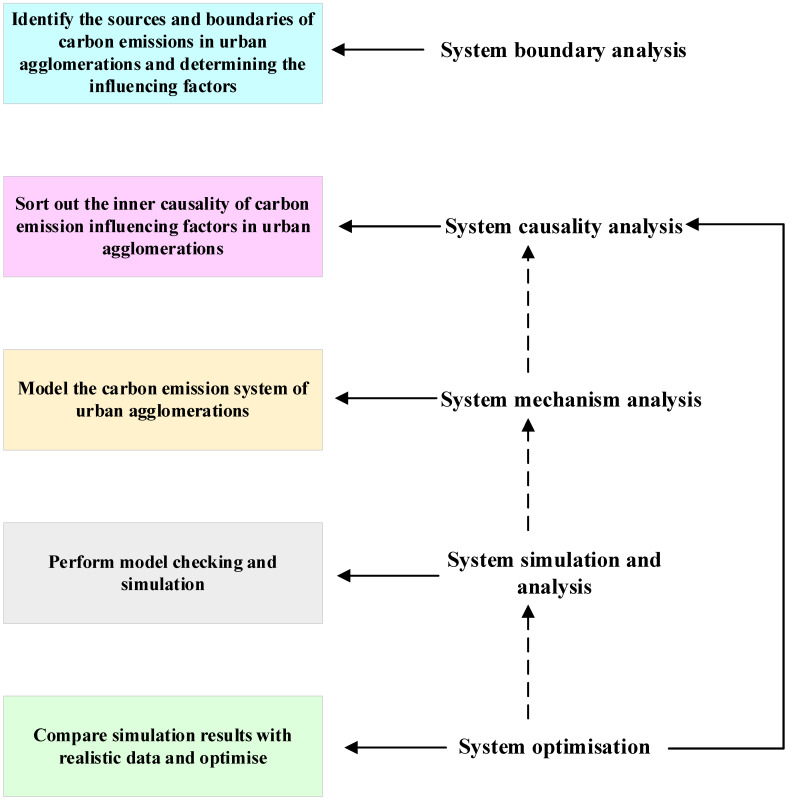
System dynamics practice process.

System dynamics (SD) is the process of modeling the elements of a social system with information feedback, exploring the relationships between the elements within the model, and analysing the patterns of change [46]. Forrester has addressed the issue of the practical application of system dynamics at a methodological level and has summarised five steps for the application of system dynamics [47]. The research process of this paper is shown in Figure 2: Firstly, the system boundary of carbon emissions in an urban agglomeration needs to be clarified and the variables that have an impact on the carbon emissions of the urban agglomeration need to be identified. Secondly, we need to sort out the cause–effect relationship between the influencing factors. Third, we need to construct a feedback loop system dynamics stock and flow model to reveal the relationship between the variables. Fourthly, the above model of carbon emissions from the urban agglomeration is mathematically logical to capture the interactions within the system, and simulations are carried out with the aid of computer software to analyse the future changes in carbon emissions from the urban agglomeration under the baseline scenario. Fifthly, the system dynamics “policy laboratory” function is used to assign different values to different policy variables to study the future development trend of carbon emissions under different scenarios, compare the model simulation results with real-world behaviour and activities, and optimise accordingly.

## 4. Carbon Emission System Dynamics Construction

### 4.1. System Boundary

Confirming the model boundary is the first step in modeling the system dynamics model. Referring to the current academic research on the influencing factors of carbon emissions, the main impact factors of carbon emissions in cities and urban agglomerations include economic growth, industry structure, energy consumption, population, technology innovation, and environmental regulation. Most scholars consider that economic growth is the main driver of carbon emission increase, directly affecting the carbon emission intensity from the transportation sector, electricity supply, and other sectors [48,49,50]. Technology innovation can promote the advancement of new energy technologies and the continuous reduction in usage costs, promote the efficient and clean use of coal as well as the green and low-carbon transformation, and significantly increase the proportion of clean energy represented by hydropower, wind power, nuclear energy, and photovoltaic energy, thus continuously promoting the reduction in energy intensity and the optimisation of the energy structure [51,52]. In addition, technology innovation and environmental regulation are driving the growth of low-carbon sectors such as the tertiary sector, thereby contributing to the restructuring of the industry while reducing carbon emissions [53,54]. The growth in population size will lead to an increasing demand for energy consumption, and in turn to more carbon emissions [55]. The total energy consumption is closely related to the economic growth, environmental regulation, industry structure and other factors, and the reduction in energy intensity and energy structure is influenced by other factors that will directly contribute to carbon emissions [56,57].

Based on this, we have designed the carbon emission prediction model for the Hu-Bao-O-Yu urban agglomeration that includes five subsystems: the economic subsystem, energy subsystem, transportation subsystem, population subsystem, and carbon sink subsystem (Table 2). These five subsystems are interlinked and form a causal feedback relationship. The economic subsystem determines the industrial structure of the regional economy and the structure of energy consumption, promotes investment in technology and education through economic development, promotes the expansion of afforestation and influences the rate of car ownership. The transportation and carbon sink subsystems both contribute to economic growth and have a direct impact on the reduction in carbon emissions. Changes in the total population in the population subsystem have an impact on the transportation and energy subsystems, as well as causing changes in carbon emissions. The energy subsystem acts as a bridge to the other subsystems.

The time horizon established in this paper is 2009–2035. We forecast the trend of the relevant variables for the period 2020–2035 by empirically simulating the relevant data for the period 2009–2019, with a one-year simulation step.

### 4.2. Carbon Emission Causation

**Figure 3:**
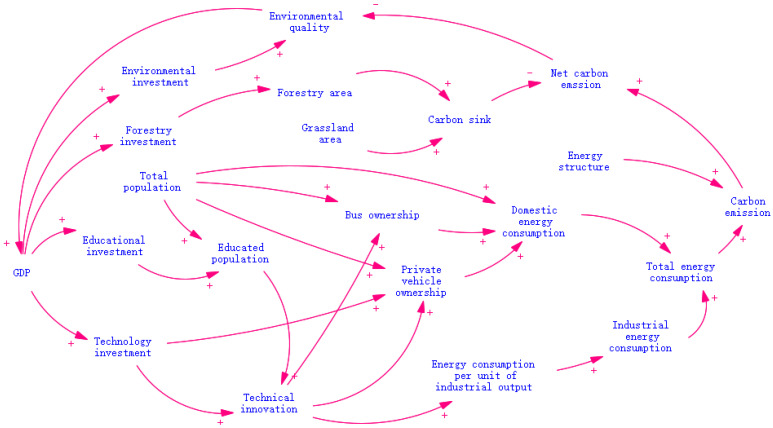
Carbon dioxide emission causation.

We summarised and refined the various subsystems involved in CO_2_ emissions in the Hu-Bao-O-Yu urban agglomeration as well as the relationships of various parameters. Based on this, we obtained the causal relationship of carbon dioxide emissions as shown in Figure 3. The figure shows that there is a complex causal relationship between the social, economic, energy, and environmental subsystems in the energy–carbon emission system.

The increase in carbon emission intensity will increase people’s investment in environmental protection and afforestation. The investment in environmental protection is conducive to improving the environmental quality, the investment in afforestation helps to increase carbon sinks, and the increase in carbon sinks has a positive effect on reducing the net carbon emissions. Increases in technology inputs reduce the energy consumption per unit of industrial output, and increases in industrial output and energy consumption per unit of output will inevitably increase industrial energy consumption; increases in the population and per capita domestic energy consumption will lead to increases in the total domestic energy consumption, and productive and domestic energy together will lead to increases in the total energy consumption and thus carbon emissions. The increase in GDP promotes the investment in technology, education, and reforestation, thus raising low carbon awareness, promoting low carbon technologies, and enhancing carbon sinks.

### 4.3. Carbon Emission System Flow Diagram

**Figure 4:**
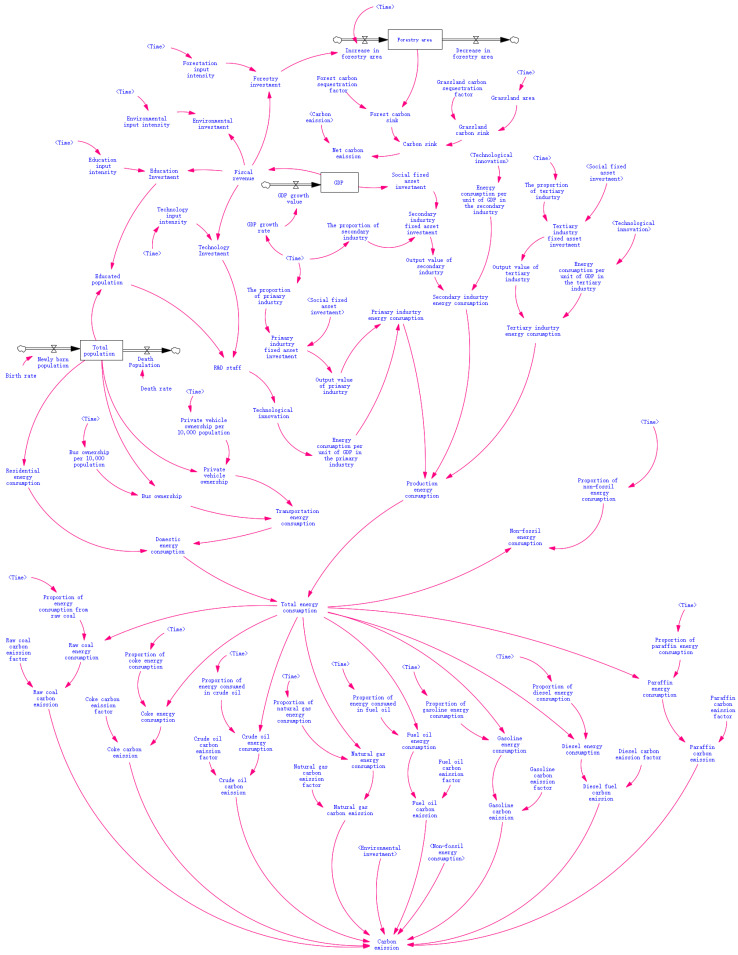
Carbon dioxide emission system flow diagram.

Based on the analysis of the sources of carbon dioxide emissions from the urban agglomeration, emission reduction methods, the interactions of influencing factors, and the selection of relevant variables, we used Vensim to draw a system causal feedback loop diagram and then determined the basic symbols in the flow diagram according to the nature of each variable in the system to draw a carbon emission system flow diagram (Figure 4). We selected a total of 89 variables in this model, including 3 state variables, 5 rate variables, and 81 auxiliary variables. Due to the limitation of space, only the main variables were selected for illustration (Table 3).

### 4.4. Data Source and Parameter Settings

The complex social, economic, demographic, environmental, and technology-related data included in the CO_2_ emission system dynamics model in this study were mainly drawn from statistics such as the China Urban Statistical Yearbook, the China Energy Statistical Yearbook, the China Transport Yearbook, as well as statistics and relevant reports from government and industry associations.

The parameters of the model were calculated by means of the ratio analysis method, the table function method, and the literature reference method. Ratio analysis refers to the derivation of quantitative relationships between variables through qualitative analysis, using selected formulae such as the energy consumption, educated population, and technology innovation. The table function method is used when the variables are in a non-linear relationship with each other. This method allows for an accurate description of the parameter changes, such as the GDP growth rate, the proportion of fixed asset investment in industry, and the proportion of non-fossil energy sources. The parameters of the fossil energy carbon emission factor, forest carbon sequestration factor, and grassland carbon sequestration factor were determined by referring to relevant literature. Due to the limitation of space, only some of the main model equations and parameter settings of Hohhot are listed (Table 4).

(1) GDP = INTEG (GDP growth value, 17,427,825)
(2) GDP growth value = GDP × GDP growth rate
(3) Total population = INTEG (Newly born population−Death population, 349)
(4) Newly born population = Total population × Birth rate
(5) Death population = Total population × Death rate
(6) Technological innovation = EXP [−13.661 + 2.17 × LN (Educated population) + 0.263 × LN (R&D staff)]
(7) R&D staff = EXP [2.176 + 0.382 × LN (Internal expenditure on R&D funding)]
(8) Educated population = EXP [−83.746 + 5.525 × LN (Total population) + 0.142 × LN (Education investment)]
(9) Forestry area = INTEG (Increase in forestry area, 39.67)
(10) Carbon sink = Forestry area × Forest carbon sequestration factor + Grassland area × Grassland carbon sequestration factor
(11) Domestic energy consumption = Total population × Residential energy consumption + Transportation energy consumption
(12) Production energy consumption = Primary industry energy consumption × Output value of primary industry + Secondary industry energy consumption × Output value of secondary industry + Tertiary industry energy consumption × Output value of tertiary industry
(13) Energy consumption = Domestic energy consumption + Production energy consumption
(14) Carbon emission = Raw coal carbon emission factor × Raw coal energy consumption + Coke carbon emission factor × Coke energy consumption + Crude oil carbon emission factor × Crude oil energy consumption + Natural gas carbon emission factor × Natural gas energy consumption + Fuel oil carbon emission factor × Fuel oil energy consumption + Gasoline carbon emission factor × Gasoline energy consumption + Diesel carbon emission factor × Diesel energy consumption + Paraffin carbon emission factor × Paraffin energy consumption
(15) Net carbon emission = Carbon emission − Carbon sink

## 5. Results and Analysis

### 5.1. Validity Test

The objective validity of the system determines the applicability and feasibility of the model. Therefore, a historical check of the validity was carried out to confirm the reasonableness and accuracy of the model before simulating the forecast. Based on the stock flow diagram of the CO_2_ emission system and the parameter settings, we applied Vensim to perform a historical simulation to check the validity of the model. The simulation interval was set to 2009–2019, with a simulation step of 1 year. If the error between the model results and the actual values was small, then the model would be well-designed and could effectively reflect the future development trend of the system. The validity of the CO_2_ emission system model was tested by running the model with all the variables in the system, based on the degree of fit between the real values of the variables and the simulation results.

As can be seen from Table 5, in the carbon dioxide emission system model, the error rate between the simulated and real values of carbon emissions for the rest of the four cities was controlled within 10%, except for Baotou, where the error was −10.72% in 2015, and Ordos and Yulin, where the errors are 11.16% and 11.97% in 2014, respectively. It can be seen that the errors of the model run results were basically controlled within 10%, the errors between the simulated and real values of the system model were small, the degree of fit was high, and the model passed the validity test. Therefore, the operation condition of the established carbon dioxide emission system dynamics model and the system parameters setting were reasonable and feasible, and the simulation results were reliable, so the model could be applied to simulate and predict the development trend of the future carbon dioxide emissions and carbon emission reduction path.

### 5.2. Carbon Dioxide Emission Simulation and Prediction

After testing the validity of the carbon dioxide emission system dynamics model of urban agglomerations, we used the actual data of 2019 as the initial variable values, and selected six indicators of the GDP growth rate, proportion of fixed asset investment in tertiary industry, proportion of energy consumption in secondary industry output, energy structure, and environmental input intensity and technology input intensity as the regulating variables from the perspectives of the economic development, industry structure, energy consumption, environmental protection, and technology innovation, and then combined the regulating variables according to their practical significance to establish six carbon emission scenarios. The specific scenario settings are shown in Table 6.

In the six scenarios, we set three rates according to the degree of change of different variables: low rate, medium rate, and high rate. The medium rate was set according to the average rate of change in the historical data in the past ten years, while the high rate and low rate were adjusted according to the requirements of the 14th Five-Year Plan and the 2035 vision of each city. The parameters of the control variables for each city and urban agglomeration are set in Table 7.

**Figure 5:**
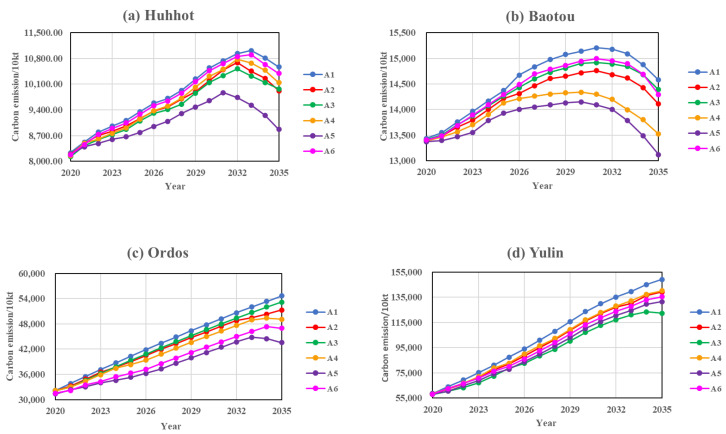
Projections of urban carbon emissions under the single regulation scenario.

**Figure 6:**
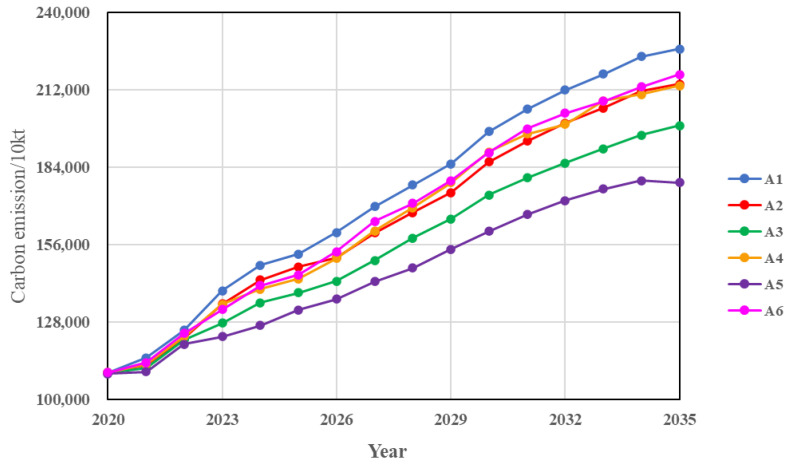
Carbon emission predications for urban agglomeration under different single regulation scenarios.

Combining the six scenarios above, we simulated the future trends and peaks of carbon emissions for each city and urban agglomeration under different scenarios, with a simulation step of 1 year. The predicted results for each city and urban agglomeration under different scenarios are shown in Figure 5 and Figure 6, and the average cumulative carbon emission reduction rates for the different scenarios compared to the base case are shown in Table 8.

(1) Baseline Scenario (A1)

As shown in Figure 5, under the baseline scenario, Hohhot and Baotou are expected to reach their carbon peaks in 2033 and 2031, with peaks of 110.213 million t (Mt) and 152.083 million t (Mt) respectively, which is consistent with the statement in the Inner Mongolia 14th Five-Year Plan and 2035 Vision of “strive to reach the peak of carbon emissions ahead of schedule”. According to the current system behaviour, the carbon emissions of Ordos and Yulin have been on an upward trend and will not reach the peak by 2035. On the whole, the Hu-Bao-O-Yu urban agglomeration will not be able to reach the peak of the carbon emissions by 2035, despite the slowdown in the increase in carbon emissions.

(2) Economic Development Scenario (A2)

There is a correlation between the economic growth and carbon emissions. We conducted a policy simulation experiment by adjusting the “GDP growth rate” parameter to take into account the pattern of slowly declining economic growth in developed regions. As shown in Figure 5 and Table 8, the average cumulative carbon emissions in Hohhot and Baotou were reduced by 2.33% and 3.15%, respectively, compared to the baseline scenario under the economic development scenario. The average cumulative carbon emissions in Ordos and Yulin fell more sharply than in the baseline scenario, by 5.65% and 5.94%, respectively, but still do not reach the peak by 2035. In summary, the average cumulative carbon emissions of the Hu-Bao-O-Yu urban agglomeration under this scenario decreased by 5.62% compared to the baseline scenario, but still cannot reach the peak by 2035, yet the growth rate of the carbon emissions has slowed down.

(3) Environmental Scenario (A3)

The increased investment in industrial pollution control can reduce carbon emissions, but there are differences in the effects on carbon emission reduction in different regions. Under the current economic development model, increasing the amount of investment in industrial pollution control will result in the best carbon emission reduction in Yulin under the single measure scenario, with the average cumulative carbon emissions decreasing by 13.27% over the projection period compared to the baseline scenario, and the city will reach its carbon peak in 2034. As shown in Figure 5 and Table 8, the other three cities’ carbon peaking times remained consistent with the baseline scenario, with the average cumulative carbon emissions decreasing by 3.76%, 1.31%, and 2.51%, respectively over the projection period compared to the baseline scenario. In summary, the average cumulative carbon emissions of the Hu-Bao-O-Yu urban agglomeration under this scenario decrease more than the baseline scenario by 9.13%, but still do not achieve peak carbon by 2035.

(4) Optimisation of Industrial Structure Scenario (A4)

The industrial structure is an important vehicle for economic operation and development, among which the tertiary sector is mostly a low-carbon sector, which is of great significance in promoting sustainable economic development. The industrial structure optimisation scenario is based on the existing economic development model, and the transfer of the secondary industry to the tertiary industry is gradually realised by appropriately increasing the proportion of fixed asset investments in the tertiary industry. In recent years, the proportion of the tertiary industry in Hohhot has been slowly increasing, and the industrial development trend is good. Therefore, the average cumulative carbon emissions under this scenario did not decrease significantly compared with the base scenario, at 2.17%, and the carbon peak time remained unchanged. Baotou’s proportion of the secondary and tertiary industry remained the same, Ordos had a higher proportion of the secondary industry than tertiary industry compared to Baotou, and Yulin had a significantly higher proportion of the secondary industry than the primary industry and tertiary industry, so the pattern of industry-led industry in the two cities remained unchanged. So, the average cumulative carbon emissions in this scenario decreased significantly compared to the baseline scenario, 7.24%, 5.93%, and 5.31% respectively, with Baotou reaching the peak one year earlier than the baseline scenario. The carbon emissions of Baotou will reach the peak one year earlier than the baseline scenario and Ordos will reach the peak in 2034, while Yulin will not reach the peak until 2035. In summary, the average cumulative carbon emissions of the Hu-Bao-E-Yu urban agglomeration under this scenario decrease by 5.81% compared to the baseline scenario, but still do not reach peak carbon by 2035.

(5) Energy Saving Scenario (A5)

The energy saving and emission reduction scenario is a scenario in which the energy intensity decreases at an accelerated rate and the share of non-fossil energy sources increase under the government’s development model of implementing a more active energy policy. As can be seen from Table 8, the average cumulative carbon emissions of the four cities under the energy saving and emission reduction scenario all show a significant decrease from the baseline scenario, at 10.37%, 8.65%, 11.71%, and 10.83% respectively. This is shown in Figure 5. Hohhot, Baotou, and Ordos reached peak carbon in 2031, 2030, and 2033 respectively under this scenario, all earlier than in the base case. Although the growth rate of the carbon emissions in Yulin decreased, it was still not possible to achieve peak carbon by 2035. In summary, the Hu-Bao-O-Yu urban agglomeration under the single scenario had the largest decrease in the average cumulative carbon emissions under this scenario compared to the base case, at 11.26%, and is expected to reach peak carbon in 2034.

(6) Low-carbon Technology Scenario (A6)

Increasing the investment in technology research and development can lead to an increase in the level of technology, which in turn reduces carbon emissions. Based on the existing economic development model, increasing the intensity of investments in technology led to an average annual decrease in the carbon emissions of 0.73% and 1.94% in Hohhot and Baotou, respectively, compared to the baseline scenario, with no change in carbon peaking time. The increase in technology investment had a greater impact on carbon emissions in Ordos and Yulin, with the average cumulative carbon emissions in this scenario decreasing by 11.20% and 8.84% in Ordos and Yulin, respectively, compared to the baseline scenario, with Ordos achieving peak carbon in 2034 and Yulin not reaching the peak by 2035, but the growth rate of the carbon emissions became slower. As shown in Figure 6 and Table 8, compared to the other single measure scenarios, low-carbon technology had a smaller effect on the carbon emission reduction in the Hu-Bao-O-Yu urban agglomeration, with an average cumulative carbon emission reduction of 4.91% compared to the baseline scenario, and was unable to achieve peak carbon by 2035.

The effect of different factors on each city and urban agglomeration under different scenarios was different. In Hohhot and Baotou, under each control scenario, there were the steep slope, buffer zone, and decline zone, which means the phase of rapid growth of carbon emissions, the phase of low and slow growth of carbon emissions, and the phase of the decline in carbon emissions. Moreover, the pattern of the carbon emission change curve was generally consistent with that of the environmental Kuznets curve. In contrast, there were few regulation scenarios in Ordos and Yulin cities and the urban agglomeration to achieve the carbon peak by 2035, and the change in the “inverted U” curve was not significant. Therefore, the impact of a single regulatory factor on the carbon emissions of urban agglomeration to reach the peak by 2035 and achieve a significant decrease was not significant.

### 5.3. Comprehensive Regulation Scenario Analysis

Based on the results of the six scenario simulations described above, it can be found that there were strong limitations if only a single parameter is adjusted. In the process of urban development and realistic development, different emission reduction policies are often not required to cooperate with each other in order to achieve the carbon emission peak target as early as possible. Based on this, we developed a comprehensive regulation of the CO_2_ emission system, simulating the changes in the system under the combined effect of different parameters, and optimising them in order to explore under which comprehensive regulation scenarios cities and urban agglomeration could achieve their carbon emission peaks early while ensuring stable economic development. We set up three comprehensive regulation scenarios, and the magnitude of the variable changes are shown in Table 9.

(1) Comprehensive Regulation Scenario (B1)

**Figure 7:**
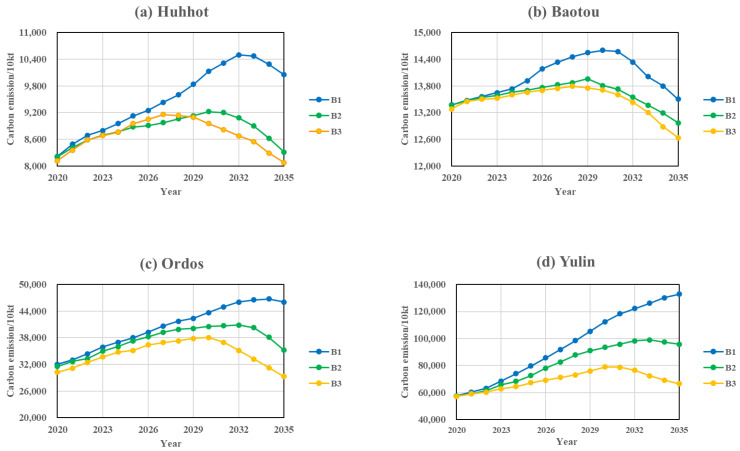
Projections of urban carbon emissions under the comprehensive regulation scenario.

**Figure 8:**
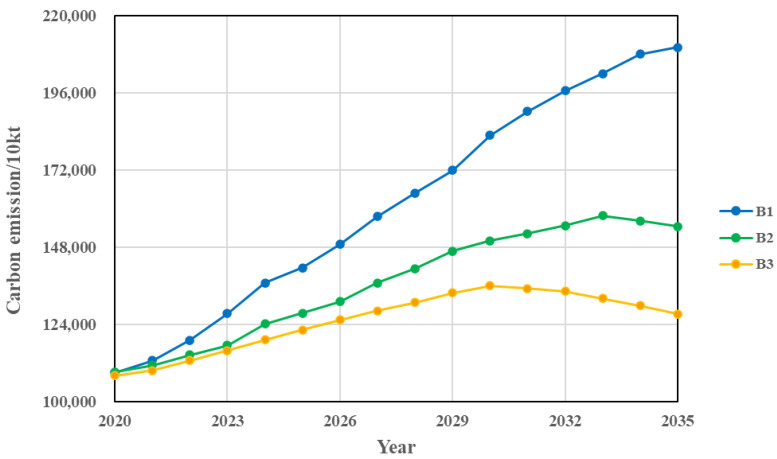
Carbon emission projections for urban agglomeration under different comprehensive regulation scenarios.

Scenario B1 is based on the economic development and industrial structure optimisation perspectives, combining the 14th Five-Year Plan of the Inner Mongolia Autonomous Region and Shaanxi Province and the 2035 Visionary Goals on GDP growth and industrial structure adjustment, setting a low GDP growth rate and a year-on-year increase in the share of the tertiary industry in Scenario B1. As shown in Figure 7 and Table 10, Hohhot and Baotou were expected to reach their carbon peaks in 2032 and 2030, respectively, under the economic and industrial optimisation scenarios, both one year earlier than the baseline scenario, and the average cumulative carbon emissions decreased by 3.91% and 10.13%, respectively, compared to the baseline scenario, so the effect of this scenario on carbon emission reduction in Baotou is more obvious. According to the behavioural pattern of the B1 scenario, Ordos and Yulin will not be able to reach the peak by 2035, but the average cumulative carbon emissions will drop more than the baseline scenario, by 8.64% and 8.91%, respectively. As shown in Figure 8 and Table 10, collectively, the Hu-Bao-O-Yu urban agglomeration under this scenario still cannot achieve peak carbon emissions by 2035, but the average cumulative carbon emissions decrease by 7.24% compared to the baseline scenario, and the growth rate of carbon emissions gradually slows down.

(2) Comprehensive Regulation Scenario (B2)

Combined with the comprehensive work plan for energy saving and emission reduction in the 14th Five-Year Plan of the four major cities, the energy structure targets of each city were added to the comprehensive control scenario B1, and the comprehensive control scenario B2 was established to explore the carbon emissions of each city and urban agglomeration under the conditions of synergistic optimisation of economic development and energy development. As shown in Figure 7 and Table 10, the average cumulative carbon emissions of each city under this scenario decreased significantly compared to the baseline scenario, by 13.76%, 15.38%, 17.31%, and 16.98% respectively. All cities achieved peak carbon emissions by 2035, with Hohhot and Baotou basically achieving the “peak carbon by 2030” target. As shown in Figure 8 and Table 10, the Hu-Bao-O-Yu urban agglomeration would reach peak carbon emissions by 2033 under this scenario, and the average cumulative carbon emissions would decrease by 16.50% compared to the baseline scenario, so the synergistic development of the economy and energy will have a significant effect on the carbon emissions of the Hu-Bao-O-Yu urban agglomeration.

(3) Comprehensive Regulation Scenario (B3)

Scenario B3 takes into account the coordinated relationship between important regulatory variables such as the economic development, industrial structure, energy intensity, energy structure, environmental protection input intensity, and technology input intensity, and is the result of the combined optimisation of Scenario B1 and B2, effectively simulating the coordinated and balanced development of each city and urban agglomeration. As shown in Figure 7 and Table 10, the average cumulative carbon emissions of each city under this scenario decreased the most significantly compared to the baseline scenario, by 16.02%, 16.77%, 22.65%, and 23.16% respectively. All cities basically achieved the “peak carbon by 2030” target, with Hohhot and Baotou cities reaching peak carbon by 2027 and 2028 earlier, and Ordos and Yulin cities both reaching peak carbon by 2030. As shown in Figure 8 and Table 10, this scenario shows that the Hu-Bao-O-Yu urban agglomeration would achieve the “peak carbon emission by 2030” target, with an average cumulative carbon emission reduction of 21.87% compared to the baseline scenario, which is the most effective scenario for carbon emission reduction at present.

## 6. Conclusions

Based on the construction of a carbon emission system dynamics model for the Hu-Bao-O-Yu urban agglomeration, this paper simulates the changes in the carbon emissions of each region under different scenarios through the single or comprehensive regulation of the economic development, industry structure, energy consumption, environmental protection input, and technology input. It also analyses the impact of different scenarios on the advancement of carbon peak time and the reduction in cumulative carbon emissions of each city and urban agglomeration and draws the following conclusions.

(1) Under the baseline scenario, Hohhot and Baotou reach peak carbon by 2033 and 2031 respectively, while other cities and the Hu-Bao-O-Yu urban agglomeration have difficulty achieving peak carbon by 2035.

(2) The effect of implementing a single control measure on carbon emissions differs from region to region. Energy consumption has the best effect on the emission reduction in Hohhot, Baotou, and Ordos, while environmental protection investment is the best factor for emission reduction in Yulin, and the weakest for emission reduction in Baotou and Ordos. Hohhot and Baotou show a complete “inverted U” curve change under the regulation of any factor, while the other two cities and urban agglomeration achieve carbon peaking by 2035 in only a few cases. Among the single regulation measures, the energy policy and environmental protection input have the most significant effect on carbon emission reduction in the Hu-Bao-O-Yu urban agglomeration, while the effect of other factors on carbon emission reduction in the urban agglomeration is relatively weak.

(3) Optimising the industrial structure and regulating the economic growth rate can slow down the growth rate of carbon emissions in the urban agglomeration, but it is still not possible to achieve the carbon peak by 2035. The synergistic optimisation of the economic and energy development has a significant effect on carbon emissions in the Hu-Bao-O-Yu urban agglomeration, and all cities and urban agglomeration can achieve the peak carbon target by 2035. A combination of the economic growth, industrial structure, energy policy, environmental protection, and technology investment has the best effect on carbon emission reduction in the cities and urban agglomeration, with the average cumulative carbon emissions in the Hu-Bao-O-Yu urban agglomeration decreasing by 21.87% compared to the baseline scenario and achieving the “2030 carbon peak” target. This shows that in the case of a slow decline in economic growth, further increasing the proportion of the tertiary industry, decreasing the energy intensity, increasing the proportion of non-fossil energy, increasing pollution control expenditure, and increasing technology investments can better achieve the carbon peak in the Hu-Bao-O-Yu urban agglomeration and effectively reduce carbon emissions in the urban agglomeration.

## 7. Policy Implications

Based on the results of the above analysis, we have created differentiated carbon peaking and reduction pathways for each of the Hu-Bao-O-Yu regions.

(1) At present, Hohhot already has the advantage of industrial technology research and development, with many research institutes and a relatively large reserve of higher talents. Therefore, it should take advantage of its talent resources in the urban agglomeration, vigorously develop its research career in line with the era of big data, continuously develop new products and technology for the development of the secondary industry in the other three cities, reduce the waste of resources, and alleviate the trend of energy resource depletion. The single regulatory measure of energy consumption and environmental protection input has the most significant effect on the carbon emission reduction in Hohhot. Based on this, Hohhot should draw on the experience of advanced pollution control at home and abroad, introduce stricter mandatory-type policy tools to apply to pollution prevention and control, improve the legal basis for source control, and continuously optimise specific incentive-based policy tools such as environmental protection taxes.

(2) Baotou, as a pioneer in this urban agglomeration, has a good energy consumption and industrial structure, but there is still much room for reduction. Strictly controlling the use of fossil energy, expanding the application of clean energy, promoting the development of the tertiary industry, and reducing the energy intensity and energy structure are of specific importance for Baotou to reach the carbon peak earlier and reduce carbon emissions. At the same time, Baotou should take the energy-consuming industry as the focus of the industrial structure adjustment, construct the industrial base of the low-carbon economy through adjusting the social and economic system, vigorously and strictly control the new production capacity of the high-energy-consuming industry, and promote the transformation and upgrading of the traditional high-energy-consuming industry such as iron and steel, petrochemical, and chemical industries, to new strategic industry.

(3) Under the current development model, it is difficult for Ordos and Yulin to achieve carbon peaking by 2035. The two cities should make comprehensive use of various regulatory measures to promote the carbon emission reduction through synergistic development. The city of Ordos has huge reserves of energy resources, so it should make full use of energy in the development of the city, increase energy conversion, reduce the damage to the environment caused by resource extraction in the development process, and effectively protect the city from environmental degradation so as to build a resource-saving city. Yulin should speed up the elimination of backward production capacity, rationalise the layout of industry, control high pollution and high emission industries such as the chemical industry, and gradually reduce the energy consumption intensity by implementing capacity replacement, mergers and acquisitions, and upgrading manufacturing. In addition, the two cities should vigorously develop their manufacturing and service industries, continue to expand into the high-end industry, and focus on the government’s role in guiding the conversion efficiency of technology, constantly optimising technology innovation policies, and improving the fit between technological inventions and industrial production.

(4) Construct a collaborative emission reduction strategy for the Hu-Bao-O-Yu urban agglomeration and strengthen the inter-regional joint prevention and control of carbon emissions. As a resource-dependent urban agglomeration, the energy structure of the agglomeration should be planned in an integrated manner, the energy policy should be reasonably laid out, the advantages of technology innovation in Hohhot should be given full play, and the optimisation and upgrading of the industrial and energy structure should be promoted with innovation as the core. At the same time, it should build a collaborative innovation mechanism for low-carbon technologies in the Hu-Bao-O-Yu urban agglomeration, actively carry out regional cooperation in the research and development of low-carbon technology projects, promote CCUS technology, promote ecological carbon sequestration, and build a comprehensive and flexible carbon emission reduction linkage mechanism together.

## 8. Future Prospects

This paper establishes a simulation model of the CO_2_ emission system and conducts policy simulations to explore the evolution of CO_2_ emissions in the Hu-Bao-O-Yu urban agglomeration, which helps to formulate scientific and effective carbon emission reductions, control schemes, and optimise the carbon peak path. However, the process of building the system dynamics model is somewhat subjective in the selection of indicators and the determination of the inter-influence relationships between them. At the same time, assumptions about the number of educated people, technological innovations, and other future technologies are based on judgments of the current scenario. Therefore, further in-depth research is needed on how to further optimise the model in combination with other methodological techniques in order to better simulate carbon peaking and carbon reduction pathways. In addition, CO_2_ emission simulation studies are not an end point of research, and how to link the simulation results more organically and closely with the choice in the carbon reduction technologies is also one of the urgent tasks to be carried out in the future.

## Figures and Tables

**Table 1:** Energy standard coal conversion factor and carbon emission factor.

Energy Type	Raw Coal	Coke Carbon	Crude Oil	Natural Gas	Fuel Oil	Gasoline	Paraffin	Diesel
Standard coal factor	0.7143	0.9714	1.4286	1.3300	1.4286	1.4714	1.4714	1.4571
Carbon emission factor	0.7559	0.8550	0.5857	0.4483	0.6185	0.5538	0.5714	0.5921

Note: Each carbon emission factor is taken from the average of carbon emission factors published by five major institutions: the US Energy Information Administration, the IPCC Guidelines for National Greenhouse Gas Emissions Inventories, the National Science Council Climate Change Program, the Chinese Academy of Engineering, and the Energy Research Institute of the National Development and Reform Commission.

**Table 2:** Subsystems and main variables.

Subsystem	Main Variable
Economic subsystem	GDP, GDP growth rate, GDP growth value, Fiscal revenue, Primary industry output, Secondary industry output, Tertiary industry output, Industry fixed asset investment, Proportion of industry fixed asset investment, Proportion of primary industry, Proportion of secondary industry, Proportion of tertiary industry, Technology investment, Education investment, Environmental investment, Forestry investment, Technology input intensity, Education input intensity, Forestation input intensity, Environmental input intensity
Transportation subsystem	Number of private vehicles per 10,000 population, Total private vehicles, Number of buses per 10,000 population, Total buses, Transportation energy consumption
Energy subsystem	Energy consumption, Energy structure, Energy carbon emission factor, Energy carbon emission, Technological innovation, Primary industry energy consumption, Secondary industry energy consumption, Tertiary industry energy consumption
Population subsystem	Total population, Newly-born population, Death population, Birth rate, Death rate, Natural population growth rate, Educated population, R&D staff, Domestic energy consumption
Carbon sink subsystem	Forestry area, Increase in forestry area, Forest carbon sequestration factor, Forest carbon sink, Grassland area, Grassland carbon sequestration factor, Grassland carbon sink

**Table 3:** Description of main variables of the model.

Main Variable	Explanation
R&D staff	Number of Research and Experimental Development staff
Educated population	Number of people with a Bachelor’s degree or above
Technology input intensity	Proportion of Internal expenditure on research and experimental development to regional GDP
Education input intensity	Proportion of expenditure on education in general higher education equivalent to regional GDP
Environmental input intensity	Proportion of investment in industrial pollution control to regional GDP
Forestation input intensity	Proportion of expenditure on afforestation to regional GDP
Technological innovation	Number of patent applications received
Energy structure	Proportion of non-fossil energy consumption to total energy consumption
Energy consumption per unit of industry output	Proportion of combined industrial energy consumption to total industrial output

**Table 4:** Important parameters and their explanations.

Parameter	Parameter Value	Unit	Explanation
GDP growth rate	{[(0,0)~(30,10)],(0,0.159),(1,0.127),(2,0.113),(3,0.110), (4,0.095),(5,0.080),(8,0.076),(10,0.040),(15,0.035), (20,0.030),(26,0.024)}	%	Self-defined function
Proportion of energy consumption in secondary industry output	{[(0,0)~(30,10)],(0,0.442),(1,0.401),(2,0.427),(3,0.401), (4,0.461),(5,0.467),(10,0.449),(15,0.409),(20,0.369),(26,0.321)}	%	Self-defined function
Proportion of fixed asset investment in tertiary industry	{[(0,0)~(30,10)],(0,0.591),(1,0.587),(2,0.586),(3,0.586), (4,0.631),(5,0.663),(8,0.741),(10,0.664),(15,0.679), (20,0.694),(26,0.712)}	%	Self-defined function
Proportion of non-fossil energy	{[(0,0)~(30,10)],(0,0.022),(1,0.023),(2,0.028),(3,0.033), (4,0.037),(5,0.041),(8,0.061),(10,0.075),(15,0.125), (20,0.175),(26,0.235)}	%	Self-defined function
Natural population growth rate	0.0468	%	China Urban Statistical Yearbook
Forest carbon sequestration factor	1.6	DMNL	IPCC Report 2006
Grassland carbon sequestration factor	1.3	DMNL	IPCC Report 2006

Note: Considering the large uncertainties in socio-economic development in the post-epidemic era, as well as the current energy structure dominated by fossil fuels such as coal and the industrial structure dominated by industrial generation, we took 2019 as the benchmark, with GDP growth decreasing by 0.1% per year, the proportion of energy consumption in the secondary industry output decreasing by 0.8% per year, the proportion of fixed asset investment in the tertiary industry increasing by 0.3% per year, and the non-fossil energy increasing by 1% per year as the baseline scenario.

**Table 5:** Model validity test results.

Year	Hohhot			Baotou		
	Real Value/10,000 t	Simulation Value/10,000 t	Error Rate/%	Real Value/10,000 t	Simulation Value/10,000 t	Error Rate/%
2009	6416.71	6128.40	−4.49	7904.959	8592.655	8.69
2010	6739.08	6512.64	−3.36	11,568.16	10,843.83	−6.26
2011	8871.32	8172.36	−7.87	9609.631	10,301.09	7.19
2012	8085.19	8509.12	5.24	12,910.46	11,622.18	−9.97
2013	8935.65	8524.39	−4.60	11,604.85	12,047.64	3.81
2014	8862.34	8819.54	−0.48	12,211.2	12,505.97	2.41
2015	8628.01	8695.85	0.78	14,670.23	13,097.14	−10.72
2016	8002.05	8354.53	4.40	12,371.99	13,195.21	6.65
2017	7398.90	7996.69	8.079	12,447.35	13,250.02	6.44
2018	7309.06	7823.41	7.03	14,158.9	13,288.52	−6.14
2019	7419.22	8035.65	8.30	13,381.29	13,315.55	−0.49
**Year**	**Ordos**			**Yulin**		
	**Real Value/10,000 t**	**Simulation Value/10,000 t**	**Error Rate/%**	**Real Value/10,000 t**	**Simulation Value / 10,000 t**	**Error Rate/%**
2009	7673.64	8318.91	8.40	8933.632	9690.379	8.47
2010	12,978.82	13,383.44	3.11	11,413.98	11,834.94	3.68
2011	12,963.76	14,111.57	8.85	16,073.73	16,127.95	0.33
2012	17,299.97	16,403.96	−5.17	19,341.84	20,576.41	6.38
2013	17,596.32	18,361.22	4.34	22,579.06	24,187.66	7.12
2014	17,347.85	19,283.99	11.16	25,870.54	28,969.39	11.97
2015	21,813.79	21,172.86	−2.93	33,744.94	33,929.65	0.54
2016	20,714.07	22,028.44	6.34	36,227.85	38,076.86	5.10
2017	22,570.58	24,651.31	9.21	43,624.93	44,419.84	1.82
2018	25,317.90	27,642.04	9.17	40,140.57	43,967.85	9.53
2019	31,341.99	30,401.22	−3.00	45,775.67	49,730.55	8.63

**Table 6:** Single regulation of carbon emissions scenario setting.

Scenario	GDP Growth Rate	Proportion of Fixed Asset Investment in Tertiary Industry	Proportion of Energy Consumption in Secondary Industry Output	Energy Structure	Environmental Input Intensity	Technology Input Intensity
Baseline scenario (A1)	Medium-speed	Medium-speed	Medium-speed	Medium-speed	Medium-speed	Medium-speed
Economic development scenario (A2)	Low-speed	Medium-speed	Medium-speed	Medium-speed	Medium-speed	Medium-speed
Environmental scenario (A3)	Medium-speed	Medium-speed	Medium-speed	Medium-speed	High-speed	Medium-speed
Optimisation of industrial structure scenario (A4)	Medium-speed	High-speed	Medium-speed	Medium-speed	Medium-speed	Medium-speed
Energy saving scenario (A5)	Medium-speed	Medium-speed	Low-speed	High-speed	Medium-speed	Medium-speed
Low-carbon technology scenario (A6)	Medium-speed	Medium-speed	Medium-speed	Medium-speed	Medium-speed	High-speed

**Table 7:** Single regulation of carbon emissions scenario parameter setting(%).

Scenario	GDP Growth Rate	Proportion of Fixed Asset Investment in Tertiary Industry	Proportion of Energy Consumption in Secondary Industry Output	Energy Structure	Environmental Input Intensity	Technology Input Intensity
Baseline scenario (A1)	−0.10/−0.10/−0.12/−0.10/−0.11	0.30/0.35/0.35/0.32/0.33	−0.80/−1.20/−1.50/−1.00/−1.30	1.00/1.50/1.50/1.20/1.30	0.50/0.75/0.75/1.00/0.75	1.20/1.20/1.20/1.50/1.28
Economic development scenario (A2)	−0.15/−0.15/−0.15/−0.15/−0.16	0.30/0.35/0.35/0.32/0.33	−0.80/−1.20/−1.50/−1.00/−1.30	1.00/1.50/1.50/1.20/1.30	0.50/0.75/0.75/1.00/0.75	1.20/1.20/1.20/1.50/1.28
Environmental scenario (A3)	−0.10/−0.10/−0.12/−0.10/−0.11	0.30/0.35/0.35/0.32/0.33	−0.80/−1.20/−1.50/−1.00/−1.30	1.00/1.50/1.50/1.20/1.30	0.75/1.00/1.00/1.25/1.00	1.20/1.20/1.20/1.50/1.28
Optimisation of industrial structure scenario (A4)	−0.10/−0.10/−0.12/−0.10/−0.11	0.40/0.50/0.50/0.45/0.46	−0.80/−1.20/−1.50/−1.00/−1.30	1.00/1.50/1.50/1.20/1.30	0.50/0.75/0.75/1.00/0.75	1.20/1.20/1.20/1.50/1.28
Energy saving scenario (A5)	−0.10/−0.10/−0.12/−0.10/−0.11	0.30/0.35/0.35/0.32/0.33	−1.10/−1.50/−1.70/−1.30/−1.40	1.20/1.80/1.80/1.50/1.58	0.50/0.75/0.75/1.00/0.75	1.20/1.20/1.20/1.50/1.28
Low-carbon technology scenario (A6)	−0.10/−0.10/−0.12/−0.10/−0.11	0.30/0.35/0.35/0.32/0.33	−0.80/−1.20/−1.50/−1.00/−1.30	1.00/1.50/1.50/1.20/1.30	0.50/0.75/0.75/1.00/0.75	1.50/1.50/1.50/1.70/1.55

**Table 8:** Reduction rate of average cumulative carbon emissions over the projection period compared to the baseline scenario under different single scenarios. (%).

Scenario	Hohhot	Baotou	Ordos	Yulin	Urban Agglomeration
Economic development scenario (A2)	−2.33	−3.15	−5.65	−5.94	−5.62
Environmental scenario(A3)	−3.76	−1.31	−2.51	−13.27	−9.13
Optimisation of industrial structure scenario (A4)	−2.17	−7.24	−5.93	−5.31	−5.81
Energy saving scenario (A5)	−10.37	−8.65	−11.71	−10.83	−11.26
Low-carbon technology scenario (A6)	−0.73	−1.94	−11.20	−8.84	−4.91

**Table 9:** Comprehensive regulation of carbon emissions scenario parameter setting (%).

Scenario	GDP Growth Rate	Proportion of Fixed Asset Investment in Tertiary Industry	Proportion of Energy Consumption in Secondary Industry Output	Energy Structure	Environmental Input Intensity	Technology Input Intensity
Comprehensive regulation scenario (B1)	−0.15/−0.15/−0.15/−0.15/−0.16	0.40/0.50/0.50/0.45/0.46	−0.80/−1.20/−1.50/−1.00/−1.30	1.00/1.50/1.50/1.20/1.30	0.50/0.75/0.75/1.00/0.75	1.20/1.20/1.20/1.50/1.28
Comprehensive regulation scenario (B2)	−0.15/−0.15/−0.15/−0.15/−0.16	0.40/0.50/0.50/0.45/0.46	−1.10/−1.50/−1.70/−1.30/−1.40	1.20/1.80/1.80/1.50/1.58	0.50/0.75/0.75/1.00/0.75	1.20/1.20/1.20/1.50/1.28
Comprehensive regulation scenario (B3)	−0.15/−0.15/−0.15/−0.15/−0.16	0.40/0.50/0.50/0.45/0.46	−1.10/−1.50/−1.70/−1.30/−1.40	1.20/1.80/1.80/1.50/1.58	0.75/1.00/1.00/1.25/1.00	1.50/1.50/1.50/1.70/1.55

**Table 10:** Reduction rate of average cumulative carbon emissions over the projection period compared to the baseline scenario under different comprehensive scenarios (%).

Scenario	Hohhot	Baotou	Ordos	Yulin	Urban Agglomeration
Comprehensive regulation scenario (B1)	−3.91	−10.13	−8.14	−8.91	−7.24
Comprehensive regulation scenario (B2)	−13.27	−15.38	−17.31	−16.98	−16.50
Comprehensive regulation scenario (B3)	−16.02	−16.77	−22.65	−23.16	−21.87

## Data Availability

Not applicable.
